# Annotations

**Published:** 1901-08-03

**Authors:** 


### Annotations.

### Medicine and Midwifery.

The question of the midwives is again agitating the medical mind, and all sorts of schemes are being proposed by which to escape the comparatively simple one of demanding that, with the exception of medical practitioners, no one shall practise midwifery unless trained, examined, licensed, and controlled. That many medical practitioners are in favour of licensing and controlling the midwives is, of course, well known; but a not inconsiderable number still seem to be under the strange impression that midwifery, somehow or other, belongs to and is a part of medicine, and that all who practise midwifery without a medical qualification are in a manner interlopers. This idea appears to be founded, or at any rate to depend for support, upon the fact that, according to the Act of 1886, every person desiring to be registered under the Medical Acts must pass a qualifying examination in midwifery as well as in medicine and surgery. But nothing is said in any of the Acts to show that registration gives any such advantage in regard to the practice of midwifery as it does in regard to the practice of either medicine or surgery. No one may hold any public medical or surgical appointment unless on the register, nor can he recover fees for medical or surgical advice or attendance, or for the performance of any operation, nor can he assume any title implying that he is recognised by law as a physician, a surgeon, a licentiate in medicine and surgery, or a practitioner in medicine, or an apothecary, under a penalty of £20. But not a word about midwifery. From a careful reading of the Acts it appears then that not only may anyone practise midwifery (as of course he can either medicine or surgery so long as he makes no false pretence in the matter) without being registered, but that in regard to midwifery there is no such prohibition of the assumption of titles as there is in regard to medicine and surgery. Clearly the Medical Acts do not make medical practitioners into midwives, or give to them any special status in regard to the practice of midwifery. The doctor, as a doctor, becomes interested in a midwifery case whenever from any cause things go wrong, and when treatment as distinct from mere attendance is required. But even then his position is not that of a superior sort of midwife, but of a medical practitioner. Undoubtedly it is often convenient and most desirable that the doctor and the midwife should be rolled into one, and thus it often happens that we find medical practitioners attending ordinary midwifery; but in so doing they merely act as midwives and not as medical practitioners. This is a convenient enough arrangement, and is not without its monetary reward; but let us have no illusions. A doctor is obliged to be skilled in midwifery, and if he chooses to pass his time as a midwifery practitioner he has a perfect right to do so. But he has no exclusive right. We do not wish to enter upon the general question as to what should be done to improve the midwife or to safeguard the parturient woman; but so much has lately been written under the idea that there is something desperately wicked in midwives advertising themselves as "L.O.S.," or as "fully trained and qualified district midwife," or even as possessing a "midwifery practice," that we think it well to point out that the assumption which so many medical practitioners have got into their heads that midwifery is part of medicine and that midwives are intruders in a field on which they have some legal claim is quite unfounded.

### The Strength of Drugs.

It has long been a moot point with some people how far the restrictions imposed by the British Pharmacopœia in regard to the composition of drugs named in it when used in compounding medicines apply to the same articles when sold over the counter to the public. Admitting, as every chemist does, that in dispensing or supplying articles ordered by physicians' prescriptions he must use Pharmacopœial preparations, it is held by some that except in regard to these matters the Pharmacopœia is without authority; and there is no doubt that a custom of the trade has grown up according to which two kinds of preparations have been kept in stock, one, the Pharmacopœial for dispensing purposes, and the other—generally in much weaker form—for sale to the public. A case, recently reported, came before the High Court of Justice, King's Bench Division, which would appear to settle the matter definitely.[1] A chemist was asked for two ounces of mercury ointment and he was supplied with some ointment in a box which was labelled "The ointment—Mercurial Poison." On analysis, however, it was found to contain only 12·5 per cent. of mercury, instead of the 48·5 per cent. which it ought to have contained according to the Pharmacopœia. The case was an appeal against a conviction by the justices, and the appellant gave evidence that the ointment supplied was a dilute preparation well known to and supplied by all retail chemists, and such as would usually be supplied to a person asking for "mercury ointment." In dismissing the appeal the judge (Mr. Justice Phillimore) said that the appellant was asked for mercury ointment, and should have sold the drug in the Pharmacopœia, or, if he was going to do what he in fact did—namely, to sell an ointment in which the mercury was about one-quarter strength—he should, if he may lawfully sell such a drug (see Section 15 of the Pharmacy Act), have explained that he was selling a weaker or diluted drug, and have so named it; and he further said that if a drug to be found in the Pharmacopœia is asked for, this drug must be supplied, and that if it is not sold with the ingredients and in the proportions prescribed by the Pharmacopœia there is at least *primâ facie* evidence that what is sold is not of the nature, substance, and quality which was demanded. This seems to make the matter clear. The next thing is to make it equally clear that the law, as here laid down, applies to non-chemists as well as to chemists. It would be intolerable that of two tradesmen dealing in the same articles, as except for scheduled poisons they often are, the one should be held to book in regard to quality while the other is allowed a free hand. Certainly the Sale of Food and Drugs Act draws no distinctions, and the judge in this decision clearly says that "if a drug to be found in the Pharmacopœia is asked for," it must be supplied according to the Pharmacopœia.

[1] Public Health, Ju'y.

### Roof Conservatories on Town Houses.

Flat roofs have never attained great popularity in England, although in some workmen's dwellings they are provided. Perhaps the reason has been partly a doubt as to their being permanently weather-tight, partly to the fact that if well made they are heavy to support and expensive to construct; moreover, in the present state of the atmosphere of London, life in the open air is not a constant joy, and horticultural pursuits on house-tops are not very attractive. A glass-roofed room or conservatory, however, would be another affair. A glass ridge and furrow roof can be made practically water-tight, it need not be heavy, and if the walls of a roof conservatory are carried to the full height, it may be well protected from the ravages of the wind. With such a roof it would be perfectly possible by a simple mechanical contrivance such as is constantly used in greenhouses, to throw the whole structure open to the air and thus while obtaining protection from the weather when this is needed, to secure all the advantages of open air when the weather is propitious. The desirability of children passing a considerable portion of their time in the open air is manifest, while unfortunately it is equally manifest that in most cases town children cannot obtain fresh air without inhaling the foulest of dust. Infinitely better would it be for a child to play about in its roof conservatory, as it could do for hours every day, than to take its perfunctory "walk" or be wheeled through the London streets at a level of only about thirty inches from the ground. We notice that at a recent meeting of the American Pediatric Society, Dr. Northrup reported that by his advice a sun-room had been built on the roof of a private house in New York, a playroom in which fresh air and sunlight can be enjoyed without dust and free from the dangers of the streets, and that the family for whom the structure was built had had the satisfaction of finding that their child who had been very delicate grew up strong and well. But our suggestion is not merely to build a playroom on the roof, but to make this glass covered room itself form the roof of the building, much as a weaving shed is made to form the roof of a mill in the textile factories in the North of England.

### Medical Officers of Fever Hospitals.

The difficulties which have recently cropped up from time to time in regard to the administration of isolation hospitals raises a serious question as to the proper and best mode of providing the medical service for these institutions. The tendency has been for sanitary committees in appointing a medical officer of health to throw in, if one may so say, along with his other duties the attendance upon the patients in the hospital. In some towns attendance upon the police and the road scrapers is also "thrown in" in the same way. But surely all this is very wrong. If patients suffering from fever are to be compulsorily removed to fever hospitals the least that can be done for them is that they shall receive the best medical attendance that the district is able to provide; while in regard to the police, considering the injuries and the violence to which they are exposed, it is most desirable that their medical officer should be one who is skilled in surgery and habituated to the performance of surgical work. But in appointing a medical officer of health medicine and surgery are apt to be but secondary considerations. Every year sanitary work tends to become more specialised, and the possession of the highest sanitary qualifications is no guarantee at all that a man is a trustworthy medical practitioner or that he is *au fait* at all the details of surgical technique. The whole arrangement is defective. Every year of good work as medical officer of health, during which a man is cut off from general practice, renders him less efficient as a practitioner, although it may greatly increase his value as a sanitary official. The two lines of work are opposed and incompatible, and the sooner the fever hospitals are officered by men chosen for their efficiency as practitioners rather than for their skill in sanitary matters the better it will be for their inmates.

### Lunacy and Crime.

While admitting that hot weather makes for irritability, one cannot regard the numerous murders and crimes of violence which have lately been recorded as mere incidents of the dog days. There is a deeper cause at work. In many cases it may be suspected, and in some it is admitted, that insanity is at the root of the mischief, and the question becomes one of very considerable urgency whether some of them are not the direct result of the present condition of the law which renders it so difficult for those who are suffering from incipient mental disorder to obtain proper treatment. On every side we see hospitals for the reception and treatment of every malady under the sun—except diseases of the mind. On every side we find a general agreement, not only among medical men, but among all men, that it is best to take a stitch in time, and that if one would be cured one should seek treatment at the earliest possible stage of one's malady. It is common sense, it is common practice, it is the way adopted in regard to all diseases—except those of the mind. In regard to these the law steps in, and, on the plea of protecting the lunatic and of preventing false imprisonment, renders treatment impossible until the mischief is done. We will undertake to say that if a patient were to walk into any hospital in London and say that he felt a strong desire to murder some one, and that he wished to be controlled, there is not one that would take him in, or indeed that would dare attempt to put him under control, unless other "facts indicating insanity" were so manifest that he could be "certified." Like the dog, which, according to the popular view, must prove that he is ferocious by biting someone before his master can be punished for not keeping him under control, so with the commencing lunatic. He must take his bite, he must commit some outrageous crime, and then he must take the consequences. If the obsession under which the crime was committed was an isolated symptom he will probably be hung, but if other "facts indicating insanity" are discovered he will be kept in comfort for the rest of his life, or, as the saying is, "during his Majesty's pleasure."

